# Electrolyte balance and muscle damage after adolescent binge alcohol use: a retrospective study

**DOI:** 10.1186/s13052-026-02280-z

**Published:** 2026-06-08

**Authors:** Isabella Hunjan, Enrico Pistritto, Antonio Corsello, Mario G. Bianchetti, Ilaria Alberti, Emilia Vassilopoulou, Carlo Agostoni, Filippo Salvini, Camilla Lavagno, Gregorio P. Milani

**Affiliations:** 1 Family Medicine Institute, Faculty of Biomedical Sciences, Università della Svizzera Italiana, Lugano, Switzerland; 2 Department of Clinical Sciences and Community Health, Università Degli Studi di, Milano, Milan, Italy; 3 Pediatric Institute of Southern Switzerland, Ospedale, San Giovanni, Bellinzona, Switzerland; 4 Pediatric Unit, Fondazione IRCCS Ca’ Granda Ospedale Maggiore Policlinico, via della Commenda 9, 20122 Milan, Italy; 5 Department of Nutritional Sciences and Dietetics, School of Health Sciences, International Hellenic University, Thessaloniki, 57400 Greece; 6 Department of Life Sciences, School of Life and Health Sciences, University of Nicosia, Nicosia, 2417 Cyprus; 7 Pediatric Emergency Department, University Children’s Hospital Zurich, Zurich, Switzerland

**Keywords:** Binge drinking, Creatine kinase, Osmolality, Potassium, Sodium

## Abstract

**Background:**

Binge alcohol drinking is a recognized cause of peripheral muscle damage, leading to increased creatine kinase levels. However, the potential role of electrolyte and osmolality changes in muscle damage remains unclear. This study investigates the prevalence of muscle damage in adolescents after binge drinking and its association with ethanol, potassium, sodium, calcium, and osmolality levels.

**Methods:**

A retrospective review of medical records from the Pediatric Emergency Department, Ca’ Granda Ospedale Maggiore Policlinico, Milan, Italy, was conducted. The study included 150 adolescents (13.2 to 18.0 years of age, female to male ratio 1.03) diagnosed with binge drinking between 2013 and 2023. Exclusion criteria encompassed muscle trauma, crush syndrome, pre-existing conditions, or medications affecting muscle metabolism and electrolyte balance. Muscle damage was defined as creatine kinase levels exceeding 1.5 times the upper limit of normal.

**Results:**

Muscle damage was identified in 61 (41%) cases, predominantly in males (female to male ratio 0.24; *p* < 0.0001). Ethanol levels (1.87 [1.57–2.30] versus 2.01 [1.61–2.36] g/L; median and interquartile range), potassium (3.6 [3.3–3.9] versus 3.7 [3.5–3.9] mmol/L), sodium (140 [139–142] versus 140 [138–142] mmol/L), total calcium (2.31 [2.27–2.37] versus 2.29 [2.25–2.31]; mmol/L), albumin-adjusted calcium (2.27 [2.22–2.32] versus 2.29 [2.18–2.31]; mmol/L), effective osmolality (286 [283–290] versus 287 [284–290] mosm/kg), and total osmolality (333 [326–342] versus 337 [326–342] mosm/kg) were similar in individuals without and with muscle damage.

**Conclusions:**

In adolescents, muscle damage is common after binge alcohol drinking, particularly in males. The pathogenesis appears to be independent of ethanol concentration, electrolyte imbalances, and osmolality changes.

## Introduction

Binge drinking is a known cause of peripheral muscle damage, resulting in the release of cellular components, particularly creatine kinase and myoglobin, into the bloodstream [1–5]. Creatine kinase levels indicate the extent of muscle damage, whereas excessive myoglobin may lead to acute kidney dysfunction [1, 4, 5]. Although severe muscle damage can manifest with myalgias, muscle weakness, or red to brown urine, many cases are asymptomatic [1, 4, 5].

The muscle damage may be attributed to several factors, including the concomitant use of licit or illicit substances with the potential to induce muscle damage, muscle trauma resulting from a fall or crush syndrome, or direct myotoxicity caused by ethanol itself [2, 4, 5]. Acute ethanol intoxication has also been associated with hypokalemia, hyponatremia, hypernatremia, hyperosmolality and hypocalcemia, all of which are recognized contributors to muscle damage [6–10].

We evaluated data from the Pediatric Emergency Department, Ca’ Granda Ospedale Maggiore Policlinico Milan, Italy, to investigate the prevalence of muscle damage linked to binge drinking, its correlation with circulating ethanol levels, and the possible pathogenic role of hypokalemia, dysnatremia, hyperosmolality, and hypocalcemia.

## Methods

### Study participants - design

We retrospectively reviewed the medical records of individuals aged 10 to 18 years who presented to the Pediatric Emergency Department of the Fondazione IRCCS Ca’ Granda Ospedale Maggiore Policlinico in Milan, Italy, with a diagnosis of binge alcohol drinking between 2013 and 2023. Eligibility criteria were: presentation to the Pediatric Emergency Department with a diagnosis of binge alcohol drinking; age 10–18 years; blood ethanol concentration ≥ 0.80 g/L [11]; information availability on demographics, blood pressure, heart rate, O₂ saturation, Glasgow Coma Scale, and biochemical measurements. Exclusion criteria [4, 5, 12, 13] were: pre-existing respiratory, neurologic, metabolic, endocrine, renal, or cardiac conditions; use of medications affecting electrolyte balance or muscle metabolism; use of illicit substances; evidence of muscle trauma or crush syndrome; and intense physical activity within 3 days before presentation.

### Laboratory methods - reference values - definitions

Venous blood samples (approximately 5 ml) were collected with minimal stasis [10, 14]. Plasma concentrations of ethanol (alcohol dehydrogenase method), creatinine (Jaffe method), urea (urease method), albumin (bromcresol purple method), creatine kinase, lactate dehydrogenase, alanine aminotransferase, and γ-glutamyl transferase (enzymatic assays), as well as total bilirubin (Jendrassik–Gróf method) and total calcium (cresolphthalein complexone method) were measured using a Cobas 8000 c702 analyzer. Whole blood analysis was performed with a GEM® Premier™ 4000 autoanalyzer for hemoglobin (optical absorbance), pH, partial carbon dioxide pressure, ionized potassium, ionized sodium (direct potentiometry), and glucose (amperometry) [10, 14].

Blood pressure was expressed in mm Hg and as age- and sex-adjusted percentiles [15]. Plasma bicarbonate was calculated from pH and partial carbon dioxide pressure [10, 14], and albumin-adjusted total calcium from calcium and albumin [10, 14]. The criteria detailed in Table 1 were used to define altered level of consciousness [10, 16], hypoxemia [10, 14], dyskalemia [10, 14], dysnatremia [10, 14], abnormal effective [10, 14] or total osmolality [10, 14], altered total or albumin-adjusted calcium [10, 14], hypoglycemia [17], acute kidney injury [18], and muscle damage [19–21].

**Table 1:** Definitions used for the present study

• **Altered level of consciousness** [10, 16]: Glasgow Coma Scale score ≤12 • **Hypoxemia** [10, 14]: O_2_-saturation ≤92% • Dyskalemia* [10, 14]: hyperkalemia ≥5.1 mmol/L, hypokalemia ≤3.4 mmol/L • **Dysnatremia** [10, 14]: hypernatremia ≥146 mmol/L, hyponatremia ≤134 mmol/L • **Altered effective osmolality** [10, 14]: 2 × sodium + glucose; mmol/L ≥296 mosm/kg, ≤274 mosm/kg • **Altered total osmolality** [10, 14]: 2 × sodium + glucose + urea + ethanol; mosm/kg. ≥301 mosm/kg, ≤279 mosm/kg
• **Altered total calcium** [10, 14]: ≥2.61 mmol/L, ≤2.19 mmol/L • **Albumin-adjusted total calcium** [10, 14]: total calcium + 0.02 × [40 − albumin] • **Hypoglycemia** [18]: glucose ≤3.3 mmol/L • **Acute kidney injury** [19]: calculated from creatinine, using age- and sex-specific reference ranges, in accordance with the Kidney Disease: Improving Global Outcomes criteria • **Muscle injury** [16, 20, 21]: creatine kinase ≥1.50 upper limit of normal (>290 U/L): • subclinical muscle damage: creatine kinase 1.60-4.90 upper limit • rhabdomyolysis: creatine kinase ≥5.00 upper limit • **Other enzyme elevations**: alanine aminotransferase >50 U/L, lactate dehydrogenase >500 U/L, γ-glutamyl transferase >50 U/L

^*^Traumatic venipuncture or delays in specimen transport or processing may cause cellular potassium release and spuriously elevated or normal levels. Therefore, values ≥5.1 or ≤4.0 mmol/L were remeasured, and the lower value used for analysis, as it most likely reflects absence of hemolysis [10, 14]

### Statistics

Categorical variables are reported as counts and were analyzed using the Fisher exact test [22]. The D’Agostino-Pearson normality test showed that age, O_2_-saturation, blood pressure, heart rate, and laboratory parameters are non-normally distributed [23]. Consequently, continuous data are presented as median (interquartile range) or as boxplots [22]. In boxplots, boxes represent the 25^th^ and the 75^th^ percentiles, the central line the median, and whiskers the 10^th^-90^th^ percentiles [24]. For five or fewer continuous data points, all individual values are reported [24].

Comparisons were performed using the Wilcoxon–Mann–Whitney test, and associations assessed by simple regression with Spearman’s correlation coefficient (rₛ) [25, 26]. A two-sided *p* < 0.05 was considered statistically significant. Analyses were conducted using GraphPad Prism 11.0.0 (GraphPad Software, San Diego, USA).

## Results

For the final analysis, 150 adolescents aged 13.2 to 18.0 years were included, with a female-to-male ratio of 1.03 (Table 2). Muscle damage was identified in 61 cases (41%): 12 of the 76 females (16%) and 49 of the 74 males (66%, *p* < 0.0001). Fifty-six of 61 cases with muscle damage showed subclinical involvement (11 females and 45 males; creatine kinase ratio 1.90 [1.77–2.10]), whereas five met criteria for rhabdomyolysis (1 female and 4 males; creatine kinase ratio 5.5, 7.2, 11, 15 and 86). None of the 61 individuals with muscle damage reported symptoms such as myalgia, muscle weakness, or red to brown urine. There was no significant difference in age, ethanol blood level, blood pressure, heart rate, Glasgow Coma Scale, O_2_-saturation, urea, albumin, lactate dehydrogenase, alanine aminotransferase, γ-glutamyl transferase, total bilirubin, acid-base balance and glucose between individuals without and with muscle damage. Individuals with muscle damage were predominantly male (female to male ratio 0.24; *p* < 0.0001) and showed slightly higher hemoglobin (*p* = 0.0002), creatinine (*p* = 0.0002), alanine aminotransferase (*p* < 0.0001) and lactate dehydrogenase (*p* = 0.0009) levels. In both groups, no cases of acute kidney injury were identified.

**Table 2:** Clinical and laboratory parameters in 150 subjects 13.2 to 18.0 years of age with an acute episodic heavy ethanol use without and with muscle damage. Results are presented either as value or as median [and interquartile range]. Parameters exhibiting statistically significant differences between groups are highlighted in bold. Comparison of creatine kinase levels and ratios between cases with and without muscle damage was not performed, as a statistically significant difference is anticipated based on the clinical definition of muscle damage

				Muscle Damage		P-value
			All	Without	With	
N			150	89	61	
Demographics						
Females : males, N			76 : 74	64 : 25	12 : 49	<0.0001
Age, years			16.4 [15.5-17.1]	16.3 [15.4-17.0]	16.5 [15.6-17.3]	0.5243
Ethanol level, g/L			1.95 [1.60-2.32]	1.87 [1.57-2.30]	2.01 [1.61-2.36]	0.3753
Blood pressure						
Systolic						
Absolute values, mm Hg			109 [100-115]	109 [101-114]	109 [99-121]	0.8052
Age corrected percentile			38 [10-75]	37 [11-74]	39 [9-77]	0.5247
Diastolic						
Absolute values, mm Hg			67 [56-74]	67 [59-74]	66 [56-73]	0.7201
Age corrected percentile			75 [51-88]	75 [55-88]	74 [48-89]	0.8173
Heart rate, beats/min			86 [75-98]	86 [75-98]	86 [76-98]	0.9445
Glasgow Coma Scale						
Value			14 [11-15]	14 [12-15]	14 [11-15]	0.8078
≤12, N			55	32	23	0.8641
O_2_-saturation						
Value, %			98 [97-100]	98 [97-100]	99 [97-100]	0.1499
Hypoxemia (≤92%)			1	1	0	>0.9999
Creatine kinase						
Level, U/L			164 [11-529]	124 [95-146]	557 [510-638]	
Creatine kinase ratio			0.56 [0.40-1.82|	0.43 [0.33-0.50]	1.92 [1.79-2.20]	
**Hemoglobin, g/L**			**136 [126-146]**	**133 [123-141]**	**141 [130-148]**	**0.0002**
**Creatinine, µmol/L**			**65 [58-74]**	**63 [56-70]**	**69 [63-78]**	**0.0002**
Acute kidney injury			0	0	0	>0.9999
Urea, mmol/L			4.2 [3.3-5.0]	4.2 [3.3-4.8]	4.5 [3.5-5.3]	0.2840
Albumin, g/L			48 [46-50]	48 [45-49]	48 [46-50]	0.2392
**Alanine aminotransferase, U/L**			**16 [13-21]**	**14 [12-17]**	**20 [16-26]**	**<0.0001**
**Lactate dehydrogenase, U/L**			**208 [185-279]**	**196 [180-236]**	**251 [194-311]**	**0.0009**
Ɣ-glutamyl transferase, U/L			10 [8-13]	9 [8-12]	11 [9-14]	0.1072
Total bilirubin, µmol/L			6 [4-9]	6 [3-9]	7 [5-9]	0.1366
pH			7.32 [7.30-7.36]	7.32 [7.31-7.36]	7.31 [7.30-7.35]	0.2502
Partial carbon dioxide pressure, kPa			6.3 [5.6-6.9]	6.1 [5.5-6.8]	6.5 [5.9-7.5]	0.1432
Bicarbonate, mmol/L			23.6 [22.1-25.2]	23.4 [21.9-24.6]	23.9 [22.9-26.3]	0.1502
Glucose						
Value, mmol/L			6.0 [5.5-6.6]	5.9 [5.5-6.6]	6.1 [5.4-6.5]	0.5817
≤3.3 mmol/L			0	0	0	>0.9999

**Fig. 1:**
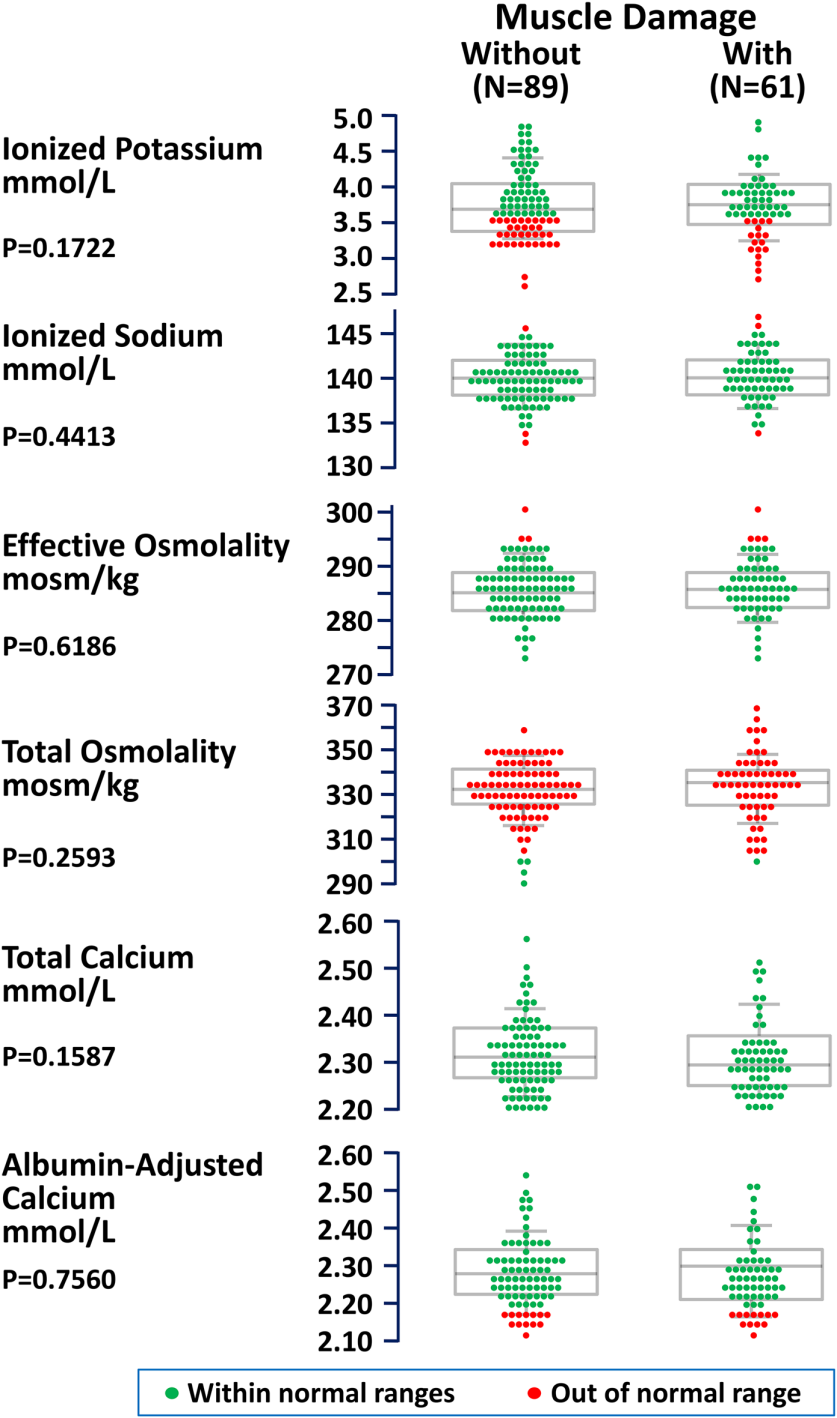
Levels of ionized potassium, ionized sodium, effective osmolality, total osmolality, total calcium and albumin-adjusted total calcium in 89 adolescents without and 61 with acute muscle damage after binge drinking. Data are shown as individual values and boxplots. In the boxplots, the box edges represent the 25^th^ and 75^th^ percentiles, the line within the box denotes the median, and the whiskers extend to the 10th and 90th percentiles. Muscle damage was defined as creatine kinase levels exceeding 1.5 times the upper limit of normal

The concentrations of potassium (3.6 [3.3–3.9] versus 3.7 [3.5–3.9] mmol/L; *p* = 0.1722), sodium (140 [139–142] versus 140 [138–142] mmol/L; *p* = 0.4413), total calcium, (2.31 [2.27–2.37] versus 2.29 [2.25–2.31]; mmol/L; *p* = 0.1587), albumin-adjusted calcium (2.27 [2.22–2.32] versus 2.29 [2.18–2.31]; mmol/L; *p* = 0.7560), as well as the effective (286 [283–290] versus 287 [284–290] mosm/kg; *p* = 0.6186) and total osmolality (333 [326–342] versus [337–326-342] mosm/kg; *p* = 0.2593) were also not statistically different between those without and with muscle damage, as shown in Fig. 1. Also, the prevalence of hypokalemia (35 out of 89 cases, corresponding to 39%, versus 17 out of 61 cases, corresponding to 28%; *p* = 0.1654), hypernatremia (1 out of 89 cases, corresponding to 1.1% versus 2 out of 61 cases, corresponding to 3.3%; *p* = 0.5667), hyponatremia (2 out of 89 cases, corresponding to 2.2%, versus 1 out of 61 cases, corresponding to 1.6%; *p* > 0.9999), effective hyperosmolality (3 out of 89 cases, corresponding to 3.4%, versus 4 out of 61 cases, corresponding to 6.6%; *p* = 0.4430), and total hyperosmolality (85 out of 89 cases, corresponding to 96%, versus 60 out of 61 cases, corresponding to 98%; *p* = 0.6487) was not statistically different between individuals without and with muscle damage. While total calcium levels were normal in all cases, some instances of hypocalcemia were observed after correction for albumin levels in both patients without and with muscle damage (13 out of 89, corresponding to 15%, versus 12 out of 61 cases, corresponding to 20%; *p* = 0.7560).

In individuals with muscle damage no significant correlation was identified between circulating sodium, potassium, effective osmolality, total osmolality, calcium or circulating ethanol, taken as independent value, and the creatine kinase ratio, taken as dependent value (Table 3). Individuals with subclinical muscle damage (*N* = 56) and those with rhabdomyolysis (*N* = 5) did not differ significantly in terms of the prevalence of hypokalemia (15 versus 2; *p* = 0.6121), dysnatremia (2 versus 1; *p* = 0.2298), albumin-adjusted hypocalcemia (11 versus 1, *p* > 0.9999) and effective (3 versus 1; 0.2962) or total (55 versus 5; *p* > 0.999) hyperosmolality.

**Table 3:** Correlations between ionized potassium, ionized sodium, effective osmolality, total osmolality, total calcium, albumin-adjusted calcium or ethanol in blood, taken as independent value, and creatine kinase ratio, taken as dependent value, in 61 individuals with muscle damage after binge drinking. The creatine kinase ratio was calculated by dividing the measured level by the corresponding upper limit of normal

Independent Value	Slope	Intercept	CorrelationCoefficient	P-value
Ionized potassium	-3.34	15.4	0.1998	0.1225
Ionized sodium	0.0270	0.519	0.0103	0.9370
Effective osmolality	0.0161	-1.34	0.0123	0.9253
Total osmolality	0.0040	1.928	0.0083	0.9492
Total calcium	-10.7	27.9	0.1183	0.3637
Albumin-adjusted calcium	0.459	2.269	0.0126	0.9234
Ethanol	-0.174	3.615	0.0279	0.9177

## Discussion

The main goal of this analysis was to assess how often a nontraumatic muscle damage occurs following binge alcohol drinking in Italian adolescents. It also aimed to determine whether ethanol level, potassium, sodium, osmolality and calcium predict muscle damage.

Creatine kinase levels are generally found pathologically elevated in one out of ten adolescents after binge drinking [26]. In a recent Dutch study, this parameter was found elevated in about one in two cases [27]. Our figures resemble those noted in the Netherlands, as indicated by the fact that creatine kinase level exceeded 1.5 times the upper limit of normal in nearly half of 150 Italian adolescents after binge alcohol drinking. Although over half of our adolescents with binge drinking were female, 80% of those with muscle damage were male. This is likely due to greater muscle mass, higher engagement in physical exertion, and a higher predisposition to genetic conditions affecting muscle integrity and stress response in males [4].

Total osmolality was almost always elevated, while effective osmolality remained normal. Potassium level was reduced in about one-third of the cases, and hyponatremia, hypernatremia, or hypocalcemia were uncommon (or even undetected). Interestingly, the occurrence of muscle damage seemed to be unrelated to the severity of total hyperosmolality, the associated potassium deficiency and ethanol level.

Total blood osmolality includes all blood solutes. Consequently, ethanol, as a small molecule, contributes to total osmolality [28, 29]. However, since ethanol freely crosses cell membranes, it does not influence water movement in or out of cells and, therefore, does not affect effective blood osmolality, which reflects the concentration of solutes exerting osmotic pressure across cell membranes [28, 29]. These observations explain why binge drinking relevantly alters total but not effective osmolality in our cases. Hypokalemia, mostly mild, is observed in about every third individual following acute ethanol intoxication [6–10]. It is hypothesized that three factors might contribute to its development: ethanol-induced diuresis, vomiting with subsequent metabolic alkalosis, and especially catecholamine release causing an intracellular potassium shift [6–10].

Diabetic hyperosmolality, both with and without acidosis, is notoriously often associated with muscle damage that correlates with the degree of hyperosmolality and hypokalemia [20]. Following binge drinking, subclinical muscle damage is neither related to the severity of hyperosmolality nor to the associated hypokalemia. Two factors likely explain this discrepancy. First, while both total and effective osmolality are elevated in diabetic hyperosmolality, only total osmolality increases following binge drinking [20, 28, 29]. Second, in diabetic hyperosmolality, potassium deficiency is typically severe and predominantly intracellular [30], whereas after binge drinking, potassium deficiency is usually mild (or even absent) and thought to be primarily extracellular [6–10]. Consequently, considering that efforts were made also to exclude causes of elevated creatine kinase such as licit and illicit substance use, muscle trauma, and crush injuries, the observed tendency towards muscle damage appears largely unexplained.

Phosphate deficiency, a known cause of muscle damage, is common in chronic alcohol use disorder [2, 3, 7, 31]. This retrospective study found no definite link between acute muscle damage after binge drinking and levels of ethanol, potassium, sodium, osmolality or calcium in previously healthy subjects. These findings support investigating phosphate depletion as a potential cause of muscle damage, despite no reported tendency toward hypophosphatemia in adolescents with acute ethanol intoxication [9]. Our results show a tendency toward hypocalcemia after adjustment of total calcium for albumin, consistent with the literature indicating that mild hypocalcemia following binge drinking is relatively common but rarely clinically significant [7]. A limitation of our study is the use of total rather than ionized calcium [32].

This study has further limitations. It is a retrospective analysis based on data from a single center. Finally, creatine kinase levels were not adjusted for pubertal stage or anthropometric variables. The main merit of the study lies in the fact that, for the first time, ethanol levels, electrolytes, and osmolality were analyzed simultaneously in 150 individuals after acute alcohol intoxication without and with muscle damage. Moreover, the laboratory techniques were particularly accurate, with electrolytes measured by direct potentiometry, as recommended by the International Federation of Clinical Chemistry and Laboratory Medicine [33].

## Conclusions

These data indicate that in adolescents with acute alcohol intoxication and an average blood ethanol concentration of approximately 2.0 g/L, muscle damage occurs in nearly half of the cases, with a markedly higher risk in males. Since no correlation was observed with ethanol or potassium levels and osmolality, a genetic susceptibility to ethanol-induced muscle damage may be hypothesized, as already established for liver disease [34].

## Data Availability

The data supporting the results of this study are available from the corresponding authors upon reasonable request.

## Declarations

### Generative artificial intelligence

Generative artificial intelligence was used to enhance the readability and language of the manuscript.
